# Sewage in Oysters

**Published:** 1881-12

**Authors:** 


### SEWAGE IN OYSTERS.

Mr. C. A. Cameron in an article entitled "On Sewage in Oysters" in the *Chemical News* states that the oysters taken from the Bay of Dublin are greatly injured and sometimes killed by the discharge of many large sewers into the Bay. The river Liffey, which a generation ago supported several kinds of edible fish, has become so polluted that these fish are now seldom seen. An examination of some oysters taken from the Bay shows that in a large proportion of them the brine had a "distinctly fœtid odor, whilst in a few cases there was a strong and unmistakable odor of sewage." "Examined microscopically, the liquid in the oysters which had a fœtid odor was found to swarm with micrococci and other low organisms similar to those usually present in sewage."

The author suggests that as water and milk may serve as vehicles for the dissemination of the germs of typhoid fever, it is possible that the same may be true of the juice found in oysters.—*Sanitary Eng'r.*

